# A Clinically-Relevant Dose of Methylphenidate Enhances Synaptic Inhibition in the Juvenile Rat Prefrontal Cortex

**DOI:** 10.17756/jrdsas.2016-030

**Published:** 2017-01-26

**Authors:** Kimberly R. Urban, Yan-Chun Li, Bo Xing, Wen-Jun Gao

**Affiliations:** 1 Department of Neurobiology and Anatomy, Drexel University College of Medicine, Philadelphia, PA 19129, USA; 2 Department of General Anesthesia, Children’s Hospital of Philadelphia, Philadelphia, PA 19104, USA

**Keywords:** Psychostimulant, Juvenile, Prefrontal cortex, GABA, Interneuron, ADHD

## Abstract

Methylphenidate (MPH) is perhaps the most commonly prescribed psychoactive substance for young children and adolescents; however, its effects on the immature brain are not well understood. MPH is increasingly abused by adolescents and prescriptions are being issued to increasingly younger children without rigorous psychological testing, raising the potential for misdiagnosis; it is therefore crucial to understand how this drug might impact a healthy, developing brain. Recently, we have shown that a clinically-relevant dose of MPH depresses the activity of pyramidal neurons in the prefrontal cortex of normal juvenile rats, but its effects on inhibitory synaptic transmission remain to be explored. We therefore recorded spontaneous (s), miniature (m), and evoked (e) inhibitory postsynaptic currents (IPSCs) in layer 5 pyramidal neurons in juvenile rat prefrontal cortex. We found a dose-dependent effect of MPH on sIPSC frequency but not amplitude, where 0.3 mg/kg significantly decreased frequency, but 1 mg/kg significantly increased frequency. Moreover, mIPSCs were not affected by either dose of MPH, whereas the amplitudes, as well as paired-pulse ratios and coefficient of variations of evoked IPSCs were significantly increased after MPH treatment, indicating a presynaptic action. Tonic GABA current was also not affected by MPH treatment. Taken together, these results suggest that MPH administration to a healthy juvenile may enhance excitation of GABAergic interneurons; thus shifting the excitation-inhibition balance in the prefrontal cortex towards inhibition, and depressing overall prefrontal cortical activity. Our findings also indicate that the adolescent brain is more sensitive to MPH than previously thought, and dose ranges need to be reconsidered for age as well as size.

## Introduction

Methylphenidate (Ritalin®, MPH) is the most commonly prescribed agent for treating attention deficit/hyperactivity disorder (ADHD) [1], a neurodevelopmental disorder that is widely recognized in both children and adults [2, 3]. MPH is also increasingly abused and taken illegally by children and teenagers to improve performance [4]. Increasing rates of prescription to younger children raise concerns about misdiagnosis and unnecessary medication, especially when the drug is prescribed as a first-line treatment or by primary care physicians without psychological testing [5]. As a psychostimulant, MPH acts primarily on the catecholamine systems to increase the concentrations of dopamine and norepinephrine in the brain, especially in the prefrontal cortex (PFC) [6], via blockade of dopamine and norepinephrine reuptake transporters [7–11].

However, both dopamine and norepinephrine act as neuromodulators to affect other neurotransmitter systems, in particular glutamate and GABA. We previously reported that a clinically-relevant dose of 1 mg/kg MPH administered via intraperitoneal injection (i.p.) to juvenile rats resulted in depression of neuronal excitability and synaptic transmission in layer 5 pyramidal neurons in the prefrontal cortex (PFC) that was dose- and age-dependent [12]. Furthermore, NMDA receptor subunit NR2B protein levels were strikingly reduced by MPH, leading to depression of short-term facilitation but augmentation of long-term potentiation in the juvenile rat prefrontal neurons [13]. These results suggest that MPH may have more wide-reaching effects on the developing brain than previously thought [4, 14, 15].

Pyramidal neurons, however, are only part of the picture of how any substance affects prefrontal cortical function. The PFC contains two major neuronal cell types: glutamatergic pyramidal neurons and GABAergic interneurons. GABAergic cells send projections to the cell bodies, axon initial segments, and dendrites of pyramidal neurons to powerfully control the output of pyramidal neurons, and thus to exert inhibitory regulation over the structures receiving projections from the pyramidal neurons. Furthermore, GABAergic interneurons in the PFC display distinct developmental changes during the juvenile and adolescent periods. There is a progressive maturation of the GABAergic system in rat mPFC in a classic “inside-out” pattern, and this involves extensive postnatal laminar changes occurring during the first 3–4 postnatal weeks, i.e., the early post-weanling period [16, 17]. There is also increased interaction of dopamine with GABA neurons during the post-weanling period [18] and age-dependent dopamine modulation of PFC interneurons [19]. It is, therefore, important to explore the role GABA may play in the effects of MPH treatment of juveniles, as it is likely that altering levels of dopamine via stimulant treatment may particularly affect its interactions with GABA during the post-weanling period, resulting in alterations in GABA signaling and maturation. In this follow-up study, we administered MPH to the juvenile rat and then recorded synaptic inhibition of pyramidal neurons in PFC. We found that MPH exerted a significant effect on GABAergic interneurons by directly affecting sIPSC frequency but not amplitude in a dose-dependent manner, as well as increasing eIPSC amplitude, paired-pulse ratio (PPR) and coefficient of variations. However, MPH did not affect mIPSCs, suggesting a presynaptic action on GABAergic inhibition.

## Materials and Methods

### Animal care and MPH treatment

Male and female Sprague-Dawley pup rats at 12 days of age (P12) were purchased from Charles River Laboratory (Horsham, PA) and were housed with their mother in a litter until 20 days old and then weaned and placed two per cage. They were housed under conditions of constant temperature (21–23 °C) and humidity on a reverse 12 hour light/dark cycle with food and water available *ad libitum.* The animal procedures were performed in accordance with the National Institutes of Health (NIH) animal use guidelines and were approved by the Institutional Animal Care and Use Committee of Drexel University College of Medicine. Upon arrival, animals were allowed 5 days to acclimate before treatment began.

Rats were divided randomly into two groups: single-dose saline and single-dose MPH. Experimental animals received a single (0.3 or 1 mg/kg, i.p.) injection of MPH and were sacrificed one hour later (PD17–25). Control animals received a single injection of saline (0.9% sodium chloride) in an amount equivalent to the MPH dose given to their littermates.

### Prefrontal cortical slice preparation

Rats were anesthetized with sodium pentobarbital (Euthasol®, Virbac Animal Health) and the level of anesthesia was determined with toe pinch. Animals were quickly decapitated and the prefrontal region removed and placed in ice-cold sucrose-rich artificial cerebrospinal fluid (aCSF) containing (87 mM NaCl, 75 mM Sucrose, 25 mM NaHCO_3_, 25 mm glucose, 2.5 mM KCl, 1.25 mM NaH_2_PO_4_, 0.5 mM CaCl_2_, 7 mM MgSO_4_) and bubbled with 95% O_2_/5% CO_2_. Horizontal slices were made 300 μm thick using a Leica 1000S Vibratome (Leica Microsystems, Bannockburn, IL). The slices were collected and incubated at 35.5 °C in sucroserich aCSF bubbled with 95% O_2_/5% CO_2_ for one hour, and then stored at room temperature until being transferred to the recording chamber.

### Electrophysiological recordings

PFC slices were bathed in a heated (36–37 °C) recording chamber with aerated (95% O_2_/5% CO_2_) Ringer’s aCSF (125 mM NaCl, 25 mM NaHCO_3_, 25 mM glucose, 2.5 mM KCl, 1.25 mM NaH_2_PO_4_, 2 mM CaCl_2_, 1 mM MgCl_2_). Layer 5 PFC pyramidal neurons were located manually with the assistance of infrared differential interference contrast to visualize cell soma shape and dendrite morphology using the Zeiss upright microscope (Thornwood, NJ). Electrode pipettes were pulled using a Sutter P97 puller (Sutter Instruments, USA) and filled with a cesium-containing intracellular solution (145 mM CsCl_2_, 2 mM MgCl_2_, 2 mM Na_2_ATP, 0.5 mM Na_2_GTP, 5 mM Na_2_ phosphocreatine, 10 mM EGTA, and 10 mM HEPES). Spontaneous and evoked IPSCs were recorded in the presence of the NMDA receptor blocker AP5 (50 μM) or R,S-CPP (10 μM) and the AMPAR blocker DNQX (20 μM) were added to the bath solution. Tetrodotoxin (TTX, 0.5 μM) was added in addition to DNQX and AP5 for recording miniature IPSCs. For spontaneous and miniature IPSCs, neurons were held in voltage-clamp at −70 mV for 5 minutes, and the negative-going currents were recorded. Miniature IPSCs (mIPSCs) were recorded similarly, after allowing the sodium channel blocker TTX (0.5 μM) to wash into the bath for 5 minutes. Only neurons that formed a giga-Ohm seal without run-down were kept for analysis.

For evoked IPSCs, the neurons were held in voltage-clamp mode at −70 mV. A bipolar stimulus electrode (CBBRC75, FHC Inc., Bowdoin, ME) was placed in layer 2/3, about 200 to 300 μm from the recording in layer 5 pyramidal neurons of the PFC. A 20-Hz train of 10 pulses was given, with stimulus strength being adjusted so that the first IPSC peak was approximately 50 pA. A total of 30 sweeps of the 10-pulse protocol were recorded from each neuron and averaged.

For tonic GABA recordings, neurons were held in voltage-clamp mode at −70 mV. Spontaneous IPSCs were recorded for 5 minutes in the presence of DNQX (20 μM) and R,S-CPP (10 μM). Next, the potent GABA-A receptor blocker Gabazine (5 μM) was bathed in for 5 minutes to block phasic GABA IPSCs. The resultant baseline shift was analyzed.

### Data analysis for electrophysiology experiments

All data was analyzed using the Clampfit 9.2 software package (Axon Laboratories, Molecular Devices). The number of spontaneous and miniature IPSCs was obtained by fitting a template IPSC from each trace and inputting it to Clampfit 9.2, then running a search for all events matching the template IPSC. The resulting IPSC count was then divided by 300 (5 minutes × 60 seconds = 300 seconds) to obtain a frequency in Hz. Amplitude was measured directly in Clampfit 9.2 by measuring the distance between baseline and peak of the IPSC. Decay time was fitted with a single exponential using the standard exponential formula in Clampfit 9.2.

The amplitudes of the evoked IPSCs were measured by averaging 30 traces from the onset to peak of IPSCs using Clampfit 9.2 software (Molecular Devices). A stable baseline, which was determined by no clear change in IPSC amplitudes and no alteration in input resistance (<20%), was a prerequisite for continuous recording and further data analysis. Only recordings with stable baseline were kept for further analysis. The decay time course of IPSCs was fitted with a single exponential using the standard exponential formula in Clampfit 9.2. Student’s t-test assuming non-equal variance was run on control versus MPH-treated for each parameter. Statistical significance was held at p<0.05.

### Drugs

MPH was purchased from Sigma-Aldrich (St. Louis, MO), and diluted to a 5 mM stock solution in physiological saline. Prepared solution was stored at −20 °C in 1 ml aliquots. Tetrodotoxin, (TTX) was purchased from Abcam (Cambridge, MA) and stored in 1 mM stock at −20 °C. DNQX was purchased from Tocris (Minneapolis, MN), diluted to 20 mM stock and stored at 4 °C. R,S-CPP was purchased from Abcam (Cambridge, MA), diluted to 10 mM stock and stored at −20 °C. AP5 was purchased from Sigma-Aldrich (St. Louis, MO) and was diluted to a 50 mM stock and stored at −20 °C. Gabazine (SR-95531 hydrobromide) was purchased from Tocris (Minneapolis, MN) and was stored at −20 °C in a 2.5 mM stock.

## Results

### Single-dose acute MPH treatment results in dose-dependent change of IPSCs in layer V pyramidal neurons from juvenile rat PFC

**Figure 1:**
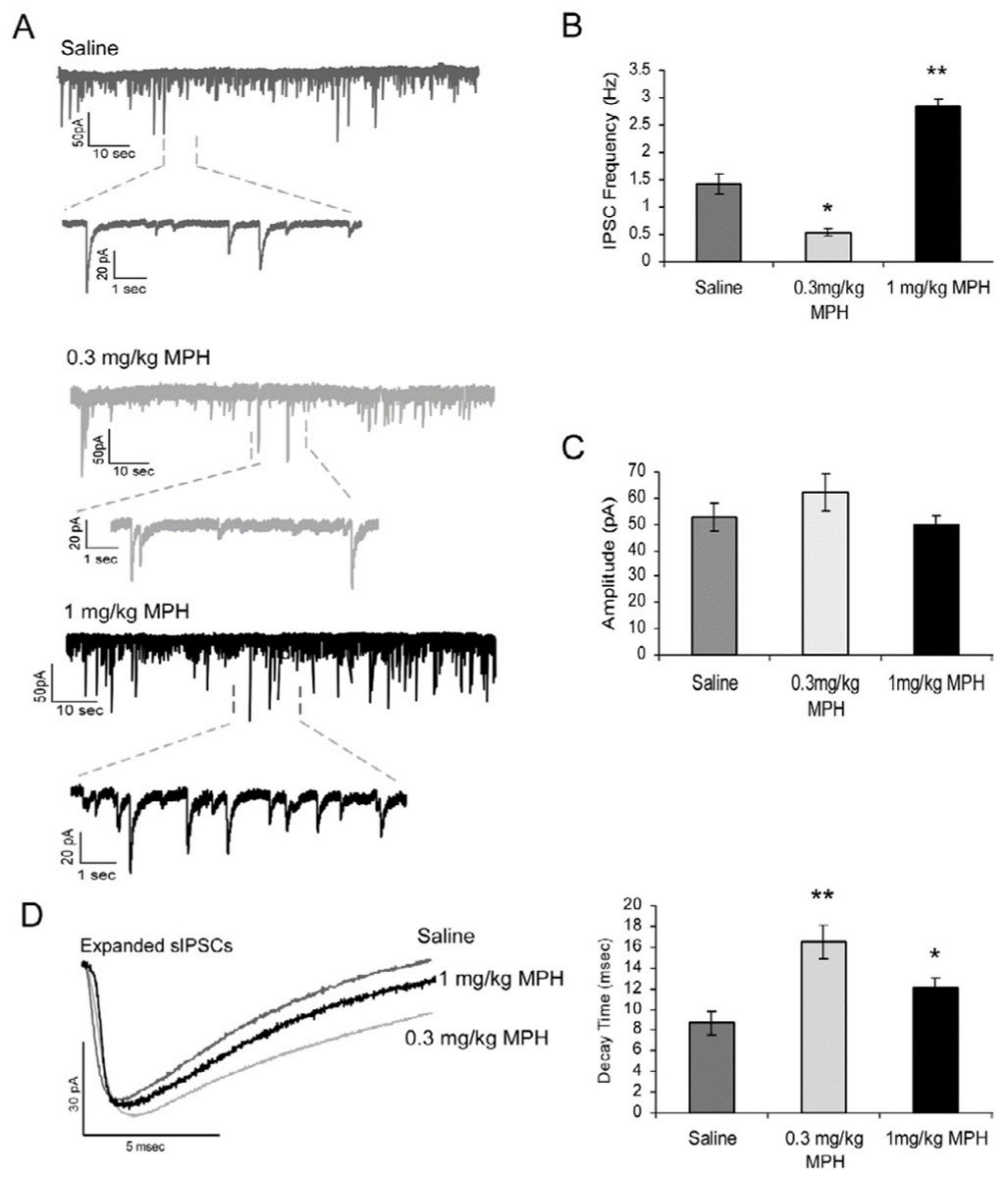
Acute treatment with MPH dose-dependently affects the frequency, but not amplitude, of sIPSCs. **A)** Representative traces of sIPSC recordings from saline-treated neurons, 0.3 mg/kg MPH-treated neurons, and 1.0 mg/kg MPH-treated neurons. **B)** The frequency of sIPSCs was significantly decreased following 0.3 mg/kg MPH (n = 12, p = 0.0002), but significantly increased following 1.0 mg/kg MPH (n = 12, p = 0.0006). **C)** The amplitudes of sIPSCs following both 0.3 mg/kg MPH (n = 12, p = 0.16) and 1.0 mg/kg MPH (n = 12, p = 0.3) were not significantly affected compared with saline control (n = 10). **D)** The sIPSC decay times were significantly increased following either dose of MPH, although to a greater degree following 0.3 mg/kg treatment (p = 0.0009) than 1.0 mg/kg treatment (p = 0.044).

In addition to excitatory pyramidal neurons, inhibitory GABAergic interneurons tightly control the neuronal activity in the PFC. In particular, GABA_A_ receptors coexist with NMDA receptors on the postsynaptic membrane, and are concurrently modulated by dopamine [20] and norepinephrine [21]. Thus, the release of dopamine and norepinephrine induced by treatment of juvenile rats with 1 mg/kg MPH may affect GABA-ergic inhibitory transmission, partially explaining the decreased excitability noted in pyramidal neurons. We hypothesized that MPH treatment would enhance GABAergic current in the juvenile PFC, resulting in an enhanced IPSC. We, therefore, examined spontaneous IPSCs in layer 5 pyramidal neurons following saline or acute MPH treatment. Following single-dose treatment with 0.3 mg/kg MPH in juvenile rats, we observed a significantly decreased frequency of spontaneous IPSCs in layer 5 pyramidal neurons (n = 12 neurons from 4 rats for saline, 10 neurons from 3 rats for MPH; p = 0.0002; Figure 1A and 1B). In contrast, following 1 mg/kg MPH treatment, sISPC frequency was significantly increased (n = 12 neurons from 4 rats for saline, 12 neurons from 4 rats for MPH, p = 0.0006; Figure 1A and 1B). The amplitude of sIPSCs was not affected by either dose of MPH (p > 0.05; Figure 1C). Interestingly, the sIPSC decay times were significantly increased by both doses, although to a stronger degree following 0.3 mg/kg than 1 mg/kg MPH (16.6 ± 1.20 ms for 0.3 mg/kg MPH and 11.8 ± 1.60 ms for 1 mg/kg MPH for 1 mg/kg MPH vs. 9.2 ± 0.95 msec for saline, p = 0.009 and p = 0.044, respectively; Figure 1D). These data suggest that MPH may dose-dependently regulate the neuronal excitability of GABAergic interneurons and the effects are likely involved in the presynaptic GABA release. Moreover, the increased sIPSC decay time suggests a potential role of GABA_B_ receptors in the effects of MPH on the GABA-mediated current because the slow component of the IPSC is usually determined by presynaptic GABA_B_ receptors [22].

### Evoked IPSCs were significantly enhanced by single-dose MPH treatment

**Figure 2:**
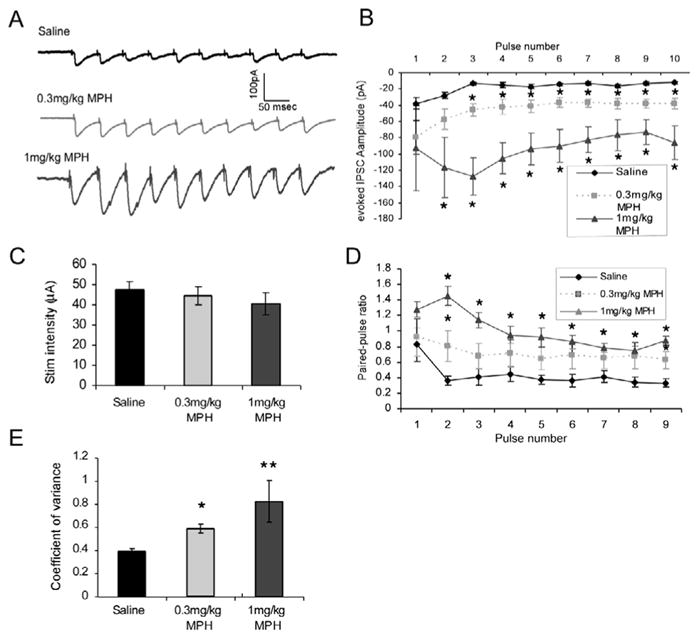
The amplitude of evoked IPSCs increased following 0.3 mg/kg and 1 mg/kg MPH. **A)** Representative traces of eIPSCs from saline, 0.3 mg/kg or 1 mg/kg MPH-treated neurons. **B)** Graphical representation of eIPSC amplitudes. The amplitudes of evoked IPSCs were significantly increased following MPH [F (2, 27), = 81.79, p = 3.5 × 10^−12^]. **C)** Stimulus intensity was not significantly different across the three treatment groups (p > 0.05). **D)** Paired-pulse ratios were significantly increased following 1 mg/kg MPH treatment [F (2, 27) = 24.87; p = 1.41 × 10^−6^] but not 0.3 mg/kg; further analysis revealed that each paired-pulse ratio was significantly increased following the 1 mg/kg MPH when compared to the equivalent saline paired-pulse ratio. Following 0.3 mg/kg MPH treatment, only the amplitudes to pulses 3 (p = 0.026) and 9 (p = 0.017) were increased. **E)** Coefficient of variances were increased by 0.3 mg/kg MPH (p = 0.021) and 1 mg/kg MPH (p = 0.0006).

To further understand the role of presynaptic and postsynaptic mechanisms in the effects of MPH on GABAergic transmission, we recorded evoked IPSCs. A stimulating electrode was placed in layer 2/3, and a recording electrode was used to patch layer 5 pyramidal neurons with the presence of selective AMPA receptor antagonist DNQX (20 μM) and NMDA receptor antagonist APV (50 μM) in the bath solution. A 10-pulse, 20-Hz train was applied to the stimulating electrode, and resultant IPSCs were recorded. We found that the amplitude of the first IPSC was significantly increased following MPH treatments (n = 7, 18.19 ± 2.62 pA for saline control versus n = 10, 45.56 ± 4.24 pA for 0.3 mg/kg MPH; p = 6.46 × 10^−8^; and versus n = 7, 94.4 ± 5.46 pA for 1.0 mg/kg MPH, p = 7.82 × 10^−7^; Figure 2A and 2B). Two-way ANOVA revealed significant difference across groups [F(2, 27) = 81.79, p = 3.5 × 10^−12^]. To confirm whether the increased IPSC amplitudes were derived from the stimulation, we measured the stimulus intensities. We found that stimulus intensities were comparable across the three treatment groups without significant differences [47.4 ± 4.3 μA for saline, 44.5 ± 4.6 μA for 0.3 mg/kg MPH, and 40.5 ± 5.7 μA for 1 mg/kg MPH; F(2, 21) = 0.435, p = 0.65; Figure 2C]. Thus, changes in the evoked IPSC amplitudes and short-term synaptic change pattern cannot be attributed to differences in stimulation intensities. In addition, paired-pulse ratios were significantly increased following 1 mg/kg MPH treatment [F(2,27) = 24.87; p = 1.41 × 10^−6^; Figure 2D], but not 0.3 mg/kg. Further inspection revealed that the overall increased paired-pulse ratios were a result of increased amplitudes of the responses to pulses 2 and 3. Paired-pulse ratio is accepted as a measure of presynaptic changes [23]. Indeed, coefficient of variance was also increased following either dose of MPH (CV = 0.393 for saline, 0.582 for 0.3 mg/kg MPH, p = 0.021, and 0.824 for 1 mg/kg MPH, p = 0.0006; Figure 2E). It seems to be contradictory that a significant decrease in frequency of spontaneous IPSCs but an increase in evoked IPSC amplitude following 0.3 mg/kg MPH treatment was observed in our animals. However, it is important to take into account the differences in these recording protocols. When we recorded spontaneous IPSCs, all inputs to the neuron are being allowed to contribute, including those from layer 2/3 as well as other layer 5 and 6 neurons, but when we recorded evoked IPSCs, we were specifically stimulating the layer 2/3 fibers; thus, inputs from layer 2/3 would be the major contributors to resulting currents. In this case, it is possible that MPH selectively excites the layer 2/3 interneurons, resulting in enhanced amplitude of evoked IPSCs. In contrast, layer 5/6 interneurons may be depressed, causing decreased sIPSC frequency. Furthermore, the alterations in paired-pulse ratio and coefficient of variance support a presynaptic effect of MPH on GABAergic transmission, suggesting excitation of the inhibitory interneurons and shifting of the excitation/inhibition balance in favor of inhibition. However, further study on this specific issue is warranted.

### Miniature IPSCs are not affected by single-dose MPH treatment

**Figure 3:**
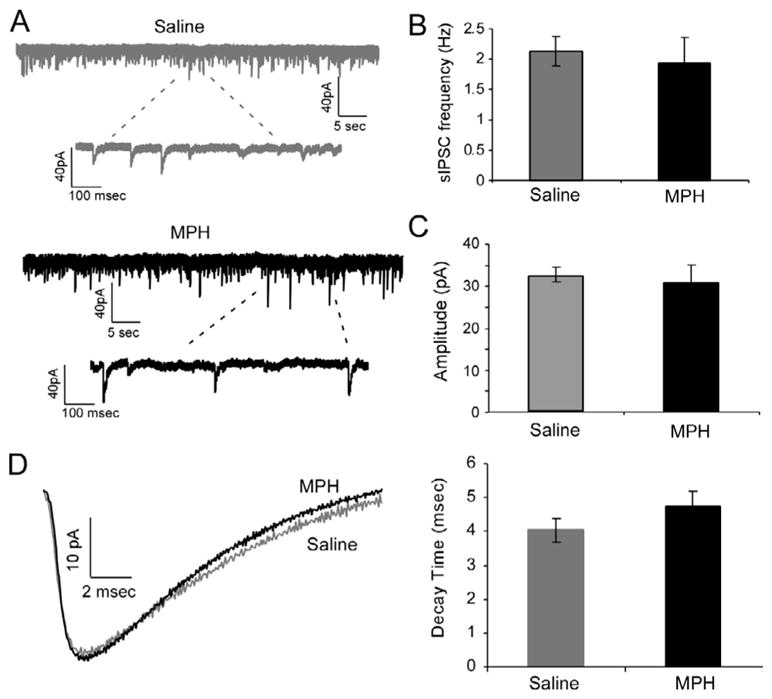
Miniature IPSCs are unchanged by MPH treatment. **A)** Representative traces from saline and 1 mg/kg MPH-treated neurons reveal similar amplitude and frequency. **B)** Frequency was unchanged by MPH treatment (p = 0.35). **C)** Amplitude of mIPSCs was unchanged by MPH treatment (p = 0.35), and **D)** Decay time was not altered (p = 0.12).

In order to further determine the role of pre- and post-synaptic changes in GABA transmission, we recorded miniature IPSCs in the presence of tetrodotoxin (0.5 μM) to block sodium channels and prevent action-potential-driven presynaptic release. Thus, only vesicular releases of GABA would occur, and any changes in frequency or amplitude of mIPSCs would more likely reveal postsynaptic GABA receptor changes. We chose to focus on 1 mg/kg MPH when exploring miniature IPSC effects, as the action of 1 mg/kg MPH appears to be more consistent. Following single-dose 1 mg/kg MPH treatment, there was no significant change in either frequency or amplitude (Figure 3), indicating that the changes seen in sIPSC and eIPSCs are indeed mediated by presynaptic changes. Therefore, the resulting enhancements in spontaneous and evoked IPSCs are likely a result of increased neuronal excitability of the inhibitory GABAergic neurons in layers 2/3 of the prefrontal cortex. Future studies will need to record directly from interneurons to determine which class(es) of interneurons are affected by MPH treatment.

### MPH treatment does not affect tonic GABA current

**Figure 4:**
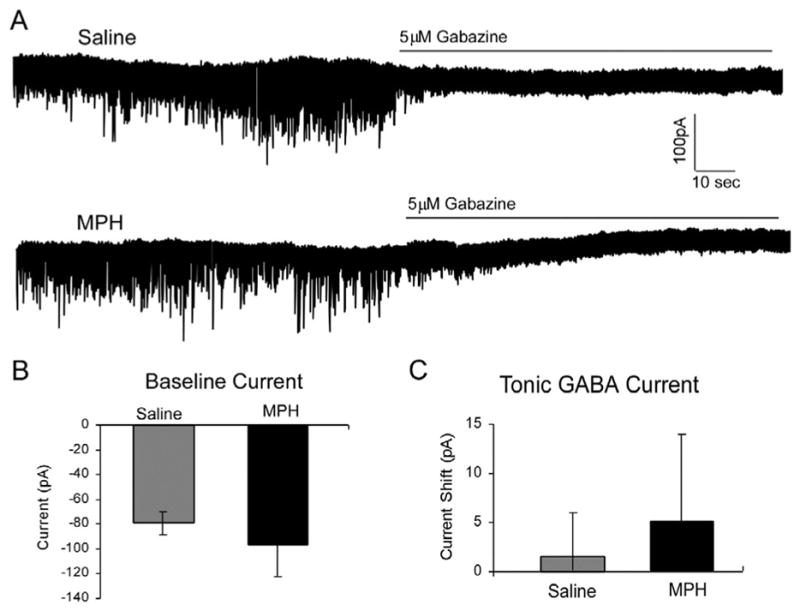
Tonic GABAergic current is not affected by MPH treatment. **A)** Representative traces showing recordings from saline control and 1 mg/kg MPH-treated animals (n = 8 saline, 7 MPH). **B)** The baseline current recorded in the first five minutes in the presence of DNQX and APV is not affected by MPH treatment (−79 + 9.3pA for saline vs. −96 + 26pA for MPH; p = 0.277). **C)** Tonic GABA current, measured as the current shift induced by the application of gabazine, is not affected by MPH treatment (1.6 + 4.45pA for saline vs. 5.06 + 8.72pA for MPH; p = 0.366).

GABA-mediated currents in the cortex consist of a phasic component that is sensitive to exogenous GABA levels, as well as a tonically-active component. The tonic component is insensitive to gabazine at levels that block miniature and spontaneous GABA-mediated IPSCs. Therefore, it can be inferred that tonic GABA-mediated current is independent of GABA_A_ receptor activity, and in PFC it is likely to be mediated by GABA_B_ receptors [24]. Since we saw an increase in the slow decay time of sIPSCs, which indicated the potential for GABA_B_ receptor involvement in MPH’s effects on inhibitory current, we examined tonic GABA-mediated currents in rats exposed to saline or MPH. Neurons were held in voltage clamp at −70 mV and a baseline activity with sIPSCs was recorded for 5 minutes in the presence of DNQX and APV to block NMDAR- and AMPAR- mediated transmission. After this baseline recording, Gabazine was infused into the bath to block all GABA_A_ receptor activity and responses to exogenous GABA (n = 8 for saline, n = 7 for MPH; Figure 4A). MPH treatment had no significant effect on either baseline current (−79 + 9.3 pA for saline vs. −96 + 26 pA for MPH; p = 0.277; Figure 4B), or the current shift induced by the introduction of 5 μM Gabazine (1.6 + 4.45 pA for saline vs. 5.06 + 8.72 pA for MPH; p = 0.366; Figure 4C). MPH treatment in juvenile rats does not appear to affect tonic GABA-mediated current; therefore, the changes seen in our IPSC frequencies are likely derived from the alterations of phasic GABA-mediated current.

## Discussion

We have shown in this study that treatment with a single dose of 1 mg/kg MPH in juvenile rats causes a significant enhancement in GABA-mediated sIPSC frequency in the layer 5 pyramidal neurons of PFC, while a lower dose of 0.3 mg/kg caused a decrease in sIPSC frequency. In addition, the amplitudes of eIPSCs were significantly increased by both doses of MPH, which are accompanied by a significant increase of PPR and CV, suggesting that the changes in IPSCs were mediated by presynaptic mechanisms. Indeed, mIPSCs, indicative of postsynaptic changes, were not altered. Furthermore, MPH treatment also did not affect the tonic GABA current.

The PFC contains both glutamatergic excitatory pyramidal neurons and GABAergic interneurons which exert inhibitory control over pyramidal neuron activity. When MPH is given systemically, as in this and our previous studies [12], it affects both cell types. Indeed, treatment with 1 mg/kg MPH resulted in an increased frequency, but not amplitude, of spontaneous IPSCs with no change in miniature IPSCs. Evoked IPSC amplitudes were significantly larger following MPH treatment, and paired-pulse ratio and CV were increased. These data suggest an activation of the inhibitory circuitry following juvenile treatment with MPH that may, in part, exacerbate or precede the decreased pyramidal neuron excitability noted in our earlier studies [12]. However, due to the increased frequency but not the amplitude of spontaneous IPSCs, combined with a lack of change to miniature currents and significantly increased PPR and CV in eIPSCs, the alterations in GABAergic current are likely derived from presynaptic mechanisms that are involved in direct effects on GABAergic interneurons and/or axonal terminals of GABAergic cells. Specifically, the interneurons are excited in a dose-dependent manner by MPH, resulting in greater release of GABA, thus increasing the frequency of sIPSCs. This finding is in agreement with a previous report, in which repeated cocaine exposure to juvenile rats increases fast-spiking interneuron excitability in the medial PFC [25].

In addition, we observed a decrease in sIPSCs and an increase in eIPSCs at a dose of 0.3 mg/kg MPH. These seemingly paradoxical findings are likely attributable to the laminar effects of MPH. It is important to note that, in spontaneous IPSC recordings, no layer-specific stimulation was given; thus, the recorded pyramidal neurons would be receiving inputs from layer 2/3 interneurons as well as layer 5 neurons. When we recorded evoked IPSCs, we specifically stimulated layer 2/3. The majority of GABAergic interneurons are located in layer 2/3 of the PFC and project inhibitory projections to the layer 5 pyramidal neurons. Thus, when we stimulated layer 2/3, we were isolating the projections leading from those layer-specific interneurons, and it is possible that MPH excites them regardless of dosage. When we recorded spontaneous IPSCs, we were allowing inputs to the pyramidal neurons from all layers; thus, connections from other layer 5 neurons could contribute to the reduced sIPSC frequency seen following 0.3 mg/kg MPH. Indeed, differential laminar effects of amphetamine on PFC parvalbumin interneurons have been reported [26], although this assumption remains to be tested.

What mechanisms could be involved in the excitation of interneurons following MPH treatment? It is currently unclear, as existing literature provides precious little data examining the effects of psychostimulants on GABAergic circuitry. What is known is that GABA agonists appear to attenuate psychostimulant abuse and self-administration, particularly with regards to cocaine. Several indirect GABA agonists (i.e. gamma-transaminase inhibitors), benzodiazepines, and GABA_B_ agonists all decrease cocaine self-administration in rodents [27]. Interestingly, our study suggests that low-dose MPH might have a GABA_B_ agonist effect; it is possible that this is the mechanism whereby low-dose MPH has been shown to reduce drug abuse and addiction in successfully treated ADHD patients [28, 29]. The presynaptic changes seen in our study may be due to GABA_B_ receptor alterations, due to the fact that the decay time was increased for spontaneous IPSCs. Specifically, slow decay time was increased; this is known to be mediated via GABA_B_ receptor activation [30]. GABA signaling has been associated with several aspects of psychostimulant-mediated behavioral changes. First, GABA_B_ receptor signaling mediates conditioned place preference and reward reinforcement. Activation of GABA_B_ receptors via the agonist baclofen has been shown to reduce cocaine self-administration as well as reinstatement of extinguished drug-seeking behavior [31–33]. Thus, inhibition of GABA_B_ receptors must play a role in the generation of psychostimulant addiction. However, how GABA_B_ receptor activation, or GABA transmission as a whole, might change in response to a low, clinically-relevant dose of MPH is likely different from the response to addiction-inducing doses of cocaine or other psychostimulants.

Still, how could application of a psychostimulant medication enhance GABAergic transmission? In our previous studies, we provided evidence that the juvenile brain is hypersensitive to MPH, and that administration of 1 mg/kg, a dose on the high end of the blood plasma-level-verified clinically-relevant dose range, produced a pattern of effects on the pyramidal neurons indicative of excessive dopamine [12, 13]. A hyperdopamine state is known to attenuate GABAergic transmission by suppressing the trafficking of GABA_A_ receptor-mediated IPSCs in pyramidal cells [20]. Therefore, if this low-dose MPH produced a simple hyperdopamine state in the juvenile brain, we would expect to see decreased IPSCs; we instead saw an increase in sIPSC frequency, eIPSC amplitude and frequency as well as an increase of paired-pulse ratio and CV, indicating a presynaptic action, as we discussed above. Thus, we must look beyond the actions of dopamine to postulate a mechanism for the effects of MPH on GABA. MPH not only raises levels of dopamine, but also norepinephrine, through blockade of the DAT and NET [34, 35]. In fact, some studies have suggested that the affinity of MPH for the NET is greater than for the DAT; thus, the drug will have a stronger effect on NE levels than on DA [36]. Despite its greater affinity for NET, MPH has been shown to cause smaller increases in NE levels than in DA levels, due to the NET having a greater affinity for DA than NE; thus, we can expect NE levels, although raised, to be raised to a milder degree than DA levels following juvenile administration of our 1 mg/kg dose [36–38]. Furthermore, NE has been shown to excite GABAergic interneurons, leading to increases in the frequency of IPSCs measured on pyramidal neurons in the hippocampus, piriform cortex, somatosensory cortex and PFC [39–43]. The effects of NE on GABAergic transmission have been shown to be mediated via an *α*-adrenoceptor activation [42, 44, 45]. However, which subtype of α-adrenoceptor (α1, α2) is responsible for the actions in PFC remains unclear [42]. It has been shown that cognitive-enhancing effects of optimal NE levels are mediated largely through α2-adrenoceptors, and that when NE increases to excessive, supraoptimal levels, α1 and β-adrenoceptors become activated and lead to impaired PFC function [46]. Does the increase in inhibitory circuitry revealed in this study indicate supra-optimal levels of NE that are impairing PFC function via excitation of interneurons that leads to depression of pyramidal cell firing, or does it demonstrate a potential mechanism whereby inhibitory activation causes sharpening of the signal-to-noise ratio by depressing unnecessary excitatory transmission? Further studies examining the effects of antagonists of α2, α1, and β-adrenoceptors could help to identify which are responsible for interneuron excitation following MPH treatment.

However, the question of how low-dose MPH treatment depresses the PFC pyramidal neuron function but excites interneuron function remains unanswered. Is the excitation of interneurons causal to the depression of pyramidal neurons? It appears that MPH effects on the two cell types are distinctly different. A recent interesting study reported that dopamine D2 receptor-mediated effects on GABAergic interneurons were not observed until early adulthood (postnatal day 50), suggesting a possible neural substrate for the maturation of dopamine-dependent prefrontal cortical functions during or after adolescence [19]. Although the complete mechanisms of how MPH affects the GABAergic inhibition remain to be explored, our findings provide additional evidence that juveniles and adolescents should be cautioned in taking MPH. It is known that during postnatal development, the dopamine system shows a progressive ingrowth of fibers into this region that continues until the early adult period [17]. In contrast, GABAergic neurons appear to complete their postnatal maturation by the early post-weaning period – juvenile and preadolescent period [16, 18]. Therefore, any psychostimulant (such as MPH) that can regulate neuronal activity, synaptic transmission, and plasticity would have the potential to alter the PFC local circuitry. We expect that alterations to GABA transmission and interneuron function would alter the excitation/inhibition (E/I) balance of the PFC, the regulation of which is critical to proper executive function and cognitive performance. This study provides novel evidence that even a single, therapeutically-relevant dose of MPH can alter interneuron signaling in the juvenile PFC. This enhancement of inhibitory transmission likely contributes to the reduced activity of excitatory pyramidal neurons we previously noted [12]. Whether these changes indicate detrimental effects or a potential therapeutic action of MPH remain to be determined; however, the overall effect appears to be dampening of PFC activation. Inactivation of PFC has been shown to impair performance on a variety of working memory and attentional tasks [47–50]. Furthermore, functional MRI imaging studies have consistently revealed reduced activation of PFC in individuals with ADHD during performance of attentional and executive function tasks [51–53]. One of the hallmarks of successful stimulant treatment is increased activation of PFC [54, 55]. Since increasing GABA transmission reduces excitability of excitatory neurons, the net effect would be reduced PFC function following a single dose of MPH in the juvenile brain, the opposite of the purported therapeutic action of MPH. This could result in impaired working memory and behavioral inhibition, along with alterations in fear learning and impaired fear extinction. However, we cannot rule out the possibility that the increased GABA transmission we noted may indicate the mechanism whereby MPH reduces drug-seeking behavior and may therefore play a part in therapeutic efficacy [27, 28, 31, 33]. Further studies are warranted to determine the cognitive and behavioral ramifications of single-dose MPH effects on GABA transmission in the juvenile PFC.

How long the changes in GABA transmission may persist following MPH treatment is another area that needs to be researched. We previously demonstrated that the reduced excitability of pyramidal neurons recovers within one week from a 3-week treatment of 1 mg/kg MPH, but not from higher doses (3 mg/kg and 9 mg/kg) [12]. If the reduced pyramidal neuron excitability is regulated in part by increased GABA transmission, then it is likely that the effects on GABA follow a similar time course, and may be partially reversible depending on dose and length of exposure.

In summary, this study provides the first evidence that even a single dose of MPH at a therapeutically-relevant level alters GABAergic signaling in the juvenile PFC in a dose-dependent manner. These results support previous findings that therapeutically-relevant doses of MPH reduce PFC activity and indicate caution may be warranted when prescribing MPH to young individuals, or when considering the drug as a cognitive enhancer to juveniles and adolescents.

## Conclusion

Methylphenidate (MPH) is a commonly prescribed psychoactive substance for young children and adolescents, but its effects on the immature brain are truly concerning. Our findings indicate that the adolescent brain is more sensitive to MPH than previously thought, and dose ranges used in clinical practice should be reconsidered for both age and body weight.

## Abbreviations

AMPA: α-amino-3-hydroxy-5-methyl-4-isoxazolepropionic acid
ADHD: Attention deficit hyperactivity disorder
APV: D-(−)-2-Amino-5-phosphonopentanoic acid
DA: Dopamine
DAT: Dopamine transporter
DNQX: 6,7-dinitroquinoxaline-2,3-dione
GABA: Gamma-aminobutyric acid
E/I: Excitation/inhibition
EPSC: Excitatory postsynaptic current
IPSC: Inhibitory postsynaptic current
MPH: Methylphenidate
NE: Norepinephrine
NET: NE norepinephrine transporter
NMDA: N-methyl-D-aspartate receptor
PFC: Prefrontal cortex
PPR: Paired-pulse ratio
TTX: Tetrodotoxin
